# Educational interventions for training oncology residents in palliative medicine: a rapid review of the available evidence to inform training standards in cancer care

**DOI:** 10.1007/s00520-026-10830-8

**Published:** 2026-06-03

**Authors:** Jayco Cheng, Irene Centeno, Barry J. A. Laird, Mari Lõhmus, Greta Chlebopaševienė, Line Foss, Stein Kaasa, Carlos Centeno

**Affiliations:** 1 ATLANTES Global Observatory of Palliative Care, Institute for Culture and Society (ICS), Universidad de Navarra, Pamplona, Spain; 2 North Estonia Medical Centre, Tallinn, Estonia; 3 Faculty of Medicine, Vilnius University, Vilnius, Lithuania; 4 Department of Oncology and Palliative Medicine, Oslo University Hospital and University of Oslo, Oslo, Norway; 5 Intensive Care Unit, Clinica Universidad de Navarra, Pamplona, España

**Keywords:** Palliative care education, Oncology residents, Clinical training, Immersive rotations, Communication skills, Competency-based training

## Abstract

**Background:**

Integrating palliative care into routine cancer care improves quality of life, communication, symptom control, and decision-making. Despite strong international recommendations, training in palliative and supportive care for oncology residents remains heterogeneous, and limited evidence informs how required competencies are best developed in clinical practice.

**Objectives:**

To synthesise the available evidence on educational interventions aimed at developing palliative and supportive care competencies among oncology residents, in order to inform training approaches relevant to contemporary cancer care, including the development of harmonised recommendations in Europe.

**Methods:**

A rapid review was conducted following PRISMA principles. PubMed was searched (2000–2025) for studies evaluating educational interventions designed to improve palliative care competencies in postgraduate medical trainees involved in oncology care. Eligible studies were synthesised narratively.

**Results:**

The search identified 509 unique records, of which 20 studies met inclusion criteria. Educational interventions were grouped into immersive (*n* = 10), involving supervised clinical practice within specialised palliative care services, and non-immersive (*n* = 10) approaches (e.g. short courses, workshops, seminars). Both types of interventions were examined in relation to internationally expected palliative care competencies for oncologists. Across studies, immersive clinical rotations—most commonly lasting 4–8 weeks—consistently improved practice-relevant competencies, including communication skills, symptom management, attitudes, knowledge, and self-efficacy. Benefits did not clearly increase with longer rotations. Mandatory immersive experiences produced broader and more consistent gains than elective formats and were more likely to influence clinical behaviours relevant to cancer care. Non-immersive interventions led to more modest but meaningful improvements, primarily in foundational knowledge, perceived preparedness, and structured communication skills. One randomised controlled trial demonstrated significant improvements in observed shared decision-making behaviours.

**Conclusions:**

Short, well-structured immersive rotations (4–8 weeks), particularly when mandatory, consistently yielded broader and more practice-relevant competency gains than elective formats. Non-immersive interventions—including workshops, online modules, and simulation—contributed meaningful improvements in foundational knowledge and structured communication skills and are best understood as complementary to, rather than substitutes for, clinical immersion. Together, these findings suggest that a tiered model combining mandatory immersive and structured non-immersive components may offer a feasible approach to strengthening PC training within oncology programmes. Findings are predominantly North American; transferability to European contexts requires further research.

**Supplementary Information:**

The online version contains supplementary material available at 10.1007/s00520-026-10830-8.

## Introduction

Palliative care (PC) is now recognised as an essential component of high-quality oncology care. When integrated early in the cancer trajectory, PC improves quality of life, symptom control, communication, decision-making, and, in some malignancies, even survival [1, 2]. International frameworks—from the World Health Organization (WHO) public health model to the new WHO Global Framework for PC Development—identify education as one of the core building blocks required to ensure timely, equitable access to PC worldwide [3]. Yet, across oncology training programmes, many residents continue to feel insufficiently prepared to manage complex symptoms, conduct goals-of-care discussions or support patients and families facing advanced disease [4]. As a result, improving palliative and supportive care education has become a strategic priority for the oncology community [5–7]. Concrete steps have included the Lancet Oncology Commission’s explicit call for embedding PC competencies into routine oncology training, the publication of ESMO and ASCO position papers defining supportive care as integral to oncology practice, and the incorporation of PC competencies into cancer centre accreditation frameworks [5, 6]. However, translating these normative positions into mandatory, evaluated curriculum requirements has proved slow and uneven, particularly in Europe, where variation in training structures, referral cultures, and institutional PC capacity adds further complexity [8]. This review focuses specifically on postgraduate medical trainees—oncology residents and fellows and internal medicine residents managing significant cancer caseloads—as the target population for the JANE-2 harmonisation initiative. Educational interventions designed for trained consultants, nursing specialists, or allied health professionals, while relevant to the broader field, involve distinct pedagogical approaches and are not addressed here.

The Lancet Commission on the integration of oncology and PC explicitly called for embedding palliative care competencies into routine cancer training [5], and the ESMO Position Paper on supportive and palliative care reaffirmed that such competencies are integral to a truly patient-centred model of oncology practice [6]. Major international societies, including MASCC and the EAPC, have likewise emphasised that education in supportive and palliative care is indispensable for delivering comprehensive cancer care [7]. Consistent with these positions, recent analyses of medical oncology education in Europe have highlighted the central role of palliative care competencies in achieving excellence and consistency in cancer care across the continent [8].

The palliative care competencies expected of oncologists are primarily established through professional consensus, clinical standards, and formal training frameworks developed by international oncology and palliative care organisations. Position papers from ESMO and ASCO, together with the Lancet Oncology Commission, consistently frame palliative care as a core component of oncology practice and outline expected competencies such as advanced communication, basic symptom management, appropriate referral to specialist palliative care, and interdisciplinary collaboration [5, 6]. These expectations are also embedded in official training curricula, European educational analyses, national specialty training programmes, and cancer centre quality and accreditation standards, where the availability and integration of palliative care are increasingly regarded as markers of comprehensive oncology care [7, 8].

Rather than emerging from comparative educational effectiveness studies, these competency expectations have largely been shaped by expert consensus, indirect clinical evidence demonstrating the benefits of palliative care integration and observed needs in routine oncology practice. Across these frameworks, there is broad convergence on a core set of competencies for oncologists, encompassing advanced communication skills—particularly shared decision-making and goals-of-care discussions—basic symptom management, appropriate attitudes towards early palliative care integration, clinical self-efficacy in end-of-life situations, effective use of palliative care resources including referral, and the ability to work within interdisciplinary teams [5, 6].

Despite these converging international expectations, palliative care education for oncology residents remains highly variable across settings, with important gaps in the structure, duration, and evaluation of training [9, 10]. To date, no comprehensive analysis has synthesised which educational interventions have been implemented, what evidence supports them, and to what extent these approaches succeed in developing the practice-relevant palliative care competencies expected of oncologists. The outcomes through which these competencies are typically assessed in the educational literature—and which frame the synthesis that follows—include gains in knowledge, shifts in attitudes towards palliative care, acquisition of communication skills in observed or simulated contexts, improvements in self-efficacy, and, where reported, changes in clinical behaviour such as referral practices and initiation of goals-of-care discussions. Immersive and non-immersive educational formats are not evaluated here as simply better or worse but as approaches likely to operate at different levels of these outcomes—a distinction that informs the interpretation of findings throughout this review.

This gap is particularly consequential in Europe, where the absence of a shared, evaluated evidence base makes it difficult for training programmes to make informed decisions about how best to develop PC competencies in oncology residents. Unlike North America, where structured PC training components have been progressively incorporated into oncology fellowship programmes over recent decades, European oncology curricula vary substantially in the extent to which palliative care is formalised, required, or evaluated [8]. Data on mandatory PC training hours within European residency programmes remain scarce, and residents’ specific educational needs in this domain are largely uncharacterised. Against this backdrop, greater harmonisation is urgently needed—and a clear evidence base is a prerequisite for that process. Within JANE-2, Work Package 6 focuses specifically on strengthening the integration of palliative care in oncology, including mapping existing educational practices and informing European recommendations for postgraduate oncology training.

### Objective

To inform the development of harmonised European guidance for integrating palliative care into oncology training, this rapid review synthesises the published evidence on educational interventions and their contribution to developing the palliative care competencies expected of oncologists.

## Method

A rapid review design was chosen as the most appropriate method for this synthesis, consistent with WHO and Cochrane guidance for evidence-informed policy development within defined timeframes [11, 12]. Systematic searching, structured appraisal, and transparent reporting were maintained throughout.

**Fig. 1:**
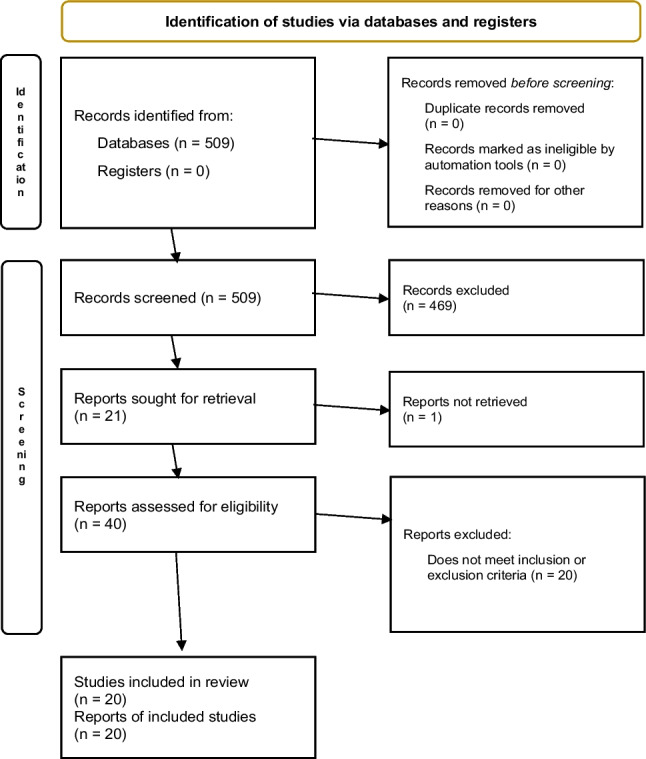
PRISMA flow diagram on palliative care education for oncology residents

The review followed core PRISMA principles [13], including predefined eligibility criteria, independent screening, transparent study selection, and structured reporting. Adaptations consistent with rapid review methodology—specifically, restriction of the search to a single database and the absence of a prospectively registered protocol—are acknowledged in the “Limitations” section (Fig. 1).

We restricted our search to PubMed, covering 1 January 2000 to 29 September 2025. The year 2000 was selected as the start date because it marks the emergence of modern competency-based medical education frameworks such as the CanMEDS 2000 [14] and the beginning of the contemporary era of structured palliative care integration into oncology training. Studies published before this date were considered unlikely to reflect current training paradigms or competency expectations. A PRISMA flow diagram is provided (Fig. 1), and the full PubMed search strategy is available in Supplementary Material (Appendix 1).

Studies were eligible if they described an educational intervention aimed at improving PC competencies in postgraduate medical trainees involved in oncology care (e.g. internal medicine residents caring for cancer patients, oncology residents or fellows), addressed clinical contexts relevant to cancer, and evaluated at least one outcome such as knowledge, communication skills, attitudes, confidence, or preparedness. Studies were excluded if they were unrelated to PC, involved fully trained oncologists or PC specialists, focused on generic communication training, took place outside oncology or serious illness care, presented curricula without evaluation, or were published before 2000.

Because oncology training pathways in many European and non-European countries include substantial rotations in internal medicine and internal medicine residents frequently provide care for hospitalised patients with advanced cancer, studies involving internal medicine trainees were included when the intervention addressed competencies directly relevant to oncology practice. This reflects both the functional proximity of internal medicine training to oncology settings and its role as a foundational stage in the training pathway of future oncologists in many national systems, where early residency includes substantial exposure to internal medicine before oncology-specific specialisation.

This is consistent with Level 2 palliative care competency expectations—including advanced communication, basic symptom management, and appropriate referral—described in the Lancet Oncology Commission framework [5].

General practitioners, by contrast, operate within distinct training structures and institutional contexts and are typically associated with Level 1 palliative care competencies. Palliative care education for GPs represents a different and important field of inquiry that falls outside the scope of this review, which focuses specifically on postgraduate trainees in oncology and oncology-adjacent settings.

Given the volume of records and the timeframe of the review, titles and abstracts were screened using a split approach: Each of the two reviewers independently screened a designated subset of records, and cases of uncertainty were discussed between the two reviewers; where consensus could not be reached, a third reviewer was consulted. This adaptation is consistent with Cochrane rapid review guidance [12].

Data extraction captured study design, trainee population, clinical setting, intervention characteristics, duration and intensity, mandatory or elective format, evaluation methods, and main outcomes. Outcomes of interest included not only knowledge, attitudes, and self-efficacy but also indicators of communication performance and clinical behaviour where reported. Substantial heterogeneity across study designs, competencies assessed, and outcome measures meant that a meta-analysis was not feasible. Therefore, findings were synthesised narratively, with immersive interventions presented first due to their direct relevance to clinical practice, followed by non-immersive interventions offering complementary insights into knowledge and skill development.

## Results

The search identified 509 unique records. After title and abstract screening, 40 full-text articles were assessed for eligibility, of which 20 met the inclusion criteria and were included in the final synthesis (Fig. 1).

Of these 20 studies on PC education for residents and fellows, ten met criteria for immersive, practice-based clinical interventions, while the remaining ten described non-immersive activities such as courses, workshops, or simulations. Of the ten immersive studies, only two were conducted in European settings: the OUTREACH study [20] and a 6-month postgraduate rotation in Switzerland [21]. Both demonstrated improvements in knowledge, attitudes, and self-efficacy broadly consistent with North American findings. However, both were conducted in university hospital contexts with relatively well-developed and accessible PC services. In many European countries, structural and cultural barriers—including limited PC specialist availability, variable referral cultures, and the absence of mandatory PC training requirements within oncology curricula—may substantially affect both the feasibility and impact of immersive rotations. These contextual differences must be considered when interpreting the transferability of predominantly North American findings to European training environments. Because immersive experiences are most directly associated with clinical competence and the integration of PC into oncology practice, this first section presents the evidence derived from these ten studies; the second part of the “Results” analyses the non-immersive interventions.

### Evidence from immersive clinical educational interventions (Table 1)

**Table 1:** Immersive educational interventions in palliative care: study designs, participants, interventions, and key outcomes for residents and oncology trainees

Author	Objective	Study design	Participants	Intervention	Evaluation tools	Key findings
Biersching (2023, Germany)	To examine whether palliative care rotations improve residents’ knowledge and self-efficacy and whether outcomes vary by rotation length or specialty	Prospective pre/post cohort; comparisons by rotation length and specialty	54 residents from multiple hospital specialties	6- or 12-month supervised inpatient PC rotations at tertiary centres First test: 4 weeks into rotation Follow-up: 4 weeks before the end of rotation	Standardised PC knowledge test; validated self-efficacy scale	Significant improvements in knowledge and self-efficacy across all durations (*p* < 0.001); no differences by rotation length or specialty; shorter rotations (6 months) produced gains equivalent to longer ones
Chang (2021, China)	To evaluate the impact of a structured palliative care training program on resident physicians’ humanistic medical skills	Randomised two-arm controlled trial	72 internal medicine residents	A brief structured program combining didactics, role-play, and 2 months of supervised clinical practice	Post-training OSCE (five competencies) and attitudes questionnaire	The intervention group showed an overall significantly improved OSCE score *p* = 0.004 compared to control
Reddy (2019, USA)	To evaluate fellows’ attitudes, beliefs, and perceived improvements in palliative care knowledge and skills after a mandatory 4-week rotation	Cross-sectional post-rotation survey. Surveys were emailed immediately after the 4 weeks and open for 6 weeks	77 haematology–oncology fellows	A mandatory 4-week inpatient and outpatient palliative care rotation with supervised clinical exposure	32-item online survey adapted from validated questionnaires	Improved perceived knowledge, skills, attitudes, and confidence; fellows rated rotation superior to others and endorsed it as mandatory. No statistical significance noted
Rossfeld (2018, USA)	To describe the experience and impact of a 4-week paediatric hospice and palliative medicine elective on residents’ self-rated competencies	Cross-sectional comparison of elective participants vs non-participants. Surveys sent 11 days after electives	124 residents (44 participants; 80 non-participants)	A 4-week paediatric hospice and palliative medicine elective with inpatient/outpatient exposure and home visits	Anonymous online survey based on ACGME competencies and PPC frameworks	Participants reported significantly higher self-rated competencies in most of the survey including communication, symptom management, psychosocial support, and EOL care compared with non-participants with *p* < 0.05
Vergo (2017, USA)	To assess acquisition of communication skills during a required 2-week palliative care rotation	Single-arm pre/post OSCE cohort (no control)	12 internal medicine residents	A required 2-week inpatient PC rotation with supervised clinical care, video modules, and pre/post OSCEs	Standardised OSCE checklist assessing interviewing, code-status discussions, and responding to emotion	Significant improvement in total OSCE score and in code-status and emotion-handling skills; residents rated the rotation highly
Centofanti (2016, Canada)	To explore residents’ experiences with end-of-life education during ICU rotations and the influence of the 3 Wishes Project	Mixed-methods single-group post-rotation study	33 residents (IM, anaesthesiology, lab medicine) who cared for 3 Wishes patients	Participation in the 3 Wishes Project providing experiential EOL care, communication, and interdisciplinary collaboration	Post-rotation semi-structured interviews and thematic analysis	Residents reported deeper empathy, improved family communication, greater comfort with EOL care, and meaningful professional/emotional growth
Olden (2009, USA)	To evaluate the impact of a required 2-week inpatient palliative care rotation on residents’ knowledge across four palliative care domains	Pre–post study with comparisons across R1, R2 (pre/post), and R3 cohorts	19 R1s; 12/17 R2s with pre/post; 20/23 R3s with 1-year follow-up	Required 2-week inpatient PC rotation including consult service, interdisciplinary meetings, seminars, and structured objectives	36-item validated PC exam across four domains (pain, non-pain symptoms, communication/ethics, terminal care)	Knowledge improved significantly during rotation (69.9% → 79.6%, *p* = 0.003), gains significant in pain and terminal care; knowledge largely retained at 1 year (76.4%)
Duong & Zulian (2006, Switzerland)	To assess whether a 6-month rotation in a specialised palliative care centre improves junior residents’ knowledge, communication skills, and attitudes towards end-of-life care	Post-rotation descriptive survey; single group, no pretest or control	33 junior residents completing a 6-month PC rotation. 4-week follow-up	A 6-month supervised clinical rotation in a specialised PC centre, including symptom management, communication, teamwork, psychological and spiritual care	Author-developed 5-point Likert questionnaire assessing perceived improvement across multiple domains	Residents reported high perceived improvement across all domains (mean 4.1–4.9/5), strongest in communication, pain control, and knowledge; 98% recommended the rotation. No statistical significance reported
Okon (2004, USA)	To evaluate whether integrating the PEACE Tool into a 4-week clinical rotation improves residents’ end-of-life knowledge and attitudes	Quasi-experimental prospective controlled study with pre/post surveys. Follow-up within 1 week of finishing rotation	18 internal medicine residents (8 intervention, 10 control)	4-week rotation using the PEACE Tool: daily symptom/decision form, PEACE booklet, pathway resources; controls received standard internal medicine training in the same PC unit	25-item questionnaire: 16-item knowledge test + 9-item attitudes scale; two-sample *t*-tests	Knowledge improved significantly in the intervention group (11.8 vs 8.1; *p* < 0.001) with positive attitudes maintained; knowledge gains comparable to didactic rotations but achieved in far less time
Liao (2004, USA)	To describe the design and early outcomes of a year-long home-hospice continuity rotation for internal medicine residents	Quasi-experimental single-group pre/post design over 1 year. Follow-up was at the last session of the year	16 internal medicine residents in two cohorts	Year-long home-hospice rotation acting as primary physicians for 6–12 patients, home visits, follow-up, case reviews, vignettes, readings, reflective projects	Pre/post 35-item knowledge/attitudes test + 360° evaluations from families, hospice teams, and faculty	Knowledge improved (+ 8.2%; *p* = 0.0175) and 360° evaluations were uniformly high; residents reported greater comfort and strong satisfaction, indicating substantial gains in communication, empathy, and real-world EOL care competence

Only ten studies offered genuine clinical immersion in PC—a surprisingly small evidence base considering the centrality of palliative competencies in modern cancer care. The geographical distribution was uneven: Seven studies originated from the USA and Canada, two from Europe (Germany and Switzerland), and one was from China. Study quality was heterogeneous. Only one randomised controlled trial (Chang et al. [15]) was identified, and only two studies (Chang et al. [15], Okon et al. [16]) incorporated a control arm. Most investigations used single-group pre/post or cross-sectional designs with small samples (typically 12–33 residents) and relied on short-term post-rotation evaluations that were often self-reported and lacked long-term follow-up. Despite these methodological limitations, the evidence consistently demonstrated that immersive exposure—whether through dedicated PC rotations, supervised clinical practice, or experiential learning within non-PC settings—enhanced residents’ knowledge, communication skills, attitudes, self-efficacy, and readiness to provide end-of-life care.

Across studies, immersive interventions varied considerably in duration and structure, although the majority involved rotations in specialised PC services. These ranged from short mandatory placements of 2 to 4 weeks to extended 6- or 12-month rotations. A short mandatory rotation of 2 weeks, as described by Olden et al. [17] in a cross-sectional study, demonstrated significant improvements in standardised knowledge test scores along with evidence of knowledge retention 1 year later (*p* value 0.003). Three 4-week rotations—the mandatory haematology–oncology fellowship rotation at MD Anderson, [18] the PEACE pathway rotation [16], and a paediatric elective at Nationwide Children’s Hospital [19]—were consistently associated with improvements in communication, symptom management, and end-of-life preparedness. Intermediate 2-month programmes, such as the structured curriculum evaluated by Chang et al. [15], showed additional benefit in objectively measured humanistic skills. Longer rotations of 6 to 12 months also improved knowledge, attitudes, and self-efficacy, although the magnitude of improvement did not clearly exceed that of shorter placements [20, 21]. Importantly, no study directly compared different rotation lengths, and the apparent similarity of outcomes across durations should be interpreted as an absence of comparative evidence rather than proof of equivalence. As a whole, the best available evidence suggests that a well-designed immersive rotation of 4 to eight 8 produces substantial educational gains.

Not all immersive interventions were based within dedicated PC units. One study evaluated a longitudinal home-hospice programme in which internal medicine residents acted as primary physicians for their hospice patients over a full academic year [22]. Another assessed the integration of a structured clinical tool—the PEACE pathway—into routine internal medicine ward work [16]. A further example, the 3 Wishes Project [23], embedded end-of-life learning within an intensive care unit, focusing on empathy, communication, and humanistic care through engagement with dying patients and their families. Despite their differences, all interventions shared a core feature: direct, supervised clinical exposure to seriously ill patients and families, often accompanied by interdisciplinary collaboration and opportunities for reflection.

The distinction between mandatory and elective formats emerged as important. Mandatory rotations tended to provide broader and more consistent benefits across entire cohorts, including those who would not have voluntarily selected PC. Studies in which all residents participated—such as Reddy et al. [18], Vergo et al. [24], and Chang et al. [15]—reported uniformly positive outcomes and strong acceptance of the rotation as an integral part of training. In contrast, elective rotations (Rossfeld et al. [19]; Duong and Zulian [21]) also demonstrated positive effects but were inherently limited by self-selection of more motivated trainees, reducing generalisability and possibly inflating perceived impact. Mandatory rotations also appeared more likely to influence actual clinical practice, including referral behaviour, suggesting a stronger potential to affect cancer care systems.

Evaluation methods varied widely. Some of the most compelling evidence came from studies using objective performance assessments, such as OSCEs [15, 24] or validated measures of PC knowledge and self-efficacy [18]. Others relied on multi-source evaluations, such as the 360° feedback used by Liao et al. [22], whereas several studies depended solely on post-rotation self-assessment, which offers useful but weaker evidence. Overall, the heterogeneity in assessment approaches and the predominance of subjective measures limited comparability across studies.

### Evidence from non-immersive clinical educational interventions (Table 2)

**Table 2:** Non-immersive educational interventions in palliative care: study designs, participants, interventions, and key findings for residents and oncology trainees

Author	Objective	Study design	Participants	Intervention	Evaluation tools	Key findings
Salas (2024, USA)	To assess feasibility and effectiveness of resident-led SIC training	Quality improvement pre–post study with 12-month follow-up	6 internal medicine residents	Condensed SIC curriculum: 90-min online didactic + 60-min virtual role-play focused on SIC documentation	Pre/post surveys + 12-month chart review	SIC training was feasible and modestly increased comfort; documented SICs rose from 4% → 15.5% over 12 months. Very small uptake limits conclusions and effect size remained modest
Hasan (2021, Canada)	To evaluate an enhanced palliative care curriculum for PHO fellows	Pre–post survey (2-month follow-up)	29 pre-courses; 25 completed courses; 8 complete post-survey	Four 2.5-h sessions: lectures, multidisciplinary panels, bereaved parent session, small-group role-plays	REDCap surveys assessing beliefs, comfort, distress, preparedness	Fellows reported improved comfort across PC domains and better coping with patient/family suffering. Positive but limited by small post-survey completion; supports interest in a national standardised PHO PC curriculum
Dewhurst (2021, UK)	To develop and pilot a PC simulation package for IM trainees	Pilot-tested pre/post confidence design	25 internal medicine trainees	Simulation-based package of eight scenarios (half-day or full-day formats)	Pre/post confidence ratings	Significant improvement in confidence across 6/7 capability domains; trainees rated scenarios feasible and useful. Demonstrates simulation as a scalable and acceptable model for PC/EOL skill-building
Nicotra (2020, USA)	To assess whether a single didactic improves understanding of palliative vs hospice care and increases consults	Pre–post educational intervention	65 internal medicine residents	30-min didactic lecture by palliative NP	Pre/post knowledge test + audit of consult volume	Knowledge rose significantly (62% → 80%, *p* < 0.001) and PC consults increased substantially (481 → 778). A brief lecture can shift awareness and referral patterns, though durability and causality remain uncertain
Henselmans (2018, Netherlands)	To evaluate whether SDM training improves oncologists’ SDM during palliative chemotherapy consultations	Randomised controlled trial with baseline and 4-month follow-up	31 oncologists (15 intervention, 16 control)	10-h SDM program: readings, two group sessions, booster with feedback, pocket card	Video-recorded standardised patient encounters + validated SDM tools	SDM performance improved markedly in the intervention group across all SDM stages; SDM OPTION12 44.1 → 64, *p* < 0.001. Provides strong evidence that structured SDM curricula enhance communication in palliative oncology
Karlen (2016, USA)	To evaluate whether a resident-led palliative care session improves comfort and attitudes	Pre–post educational intervention	95 pre-surveys; 52 post-survey 1 month after educational intervention	Single 90-min resident-led session: didactics, multimedia, role-play	Pre/post Likert surveys	Comfort increased significantly across all seven PC/EOL domains assessed. improved comfort with general knowledge of palliative medicine (*p* < 0.01), specific resources available to patients (*p* < 0.001), and explaining the difference between palliative care and end-of-life care (*p* < 0.001) Demonstrates feasibility of resident-led teaching but dependent on self-report and short-term effects
Chan (2016, USA)	To evaluate whether a multimodality ACP curriculum improves residents’ comfort and training exposure	Pre–post quality improvement study. Pre-questionnaire was 1 week before training and post was after the last observed clinic	16 PGY-2 internal medicine residents	Multicomponent ACP curriculum: online module, guidebook, 30-min didactic, simulated patient encounter with feedback, observed clinic encounter	Pre/post nine-variable questionnaires on training, comfort, attitudes	Formal ACP training and comfort improved significantly; the combined scores of all nine aspects of resident comfort in addressing ACP increased significantly from 30.7 in pre to 38.4 in post, *p* = 0.005. Well-received and suggests ACP curricula can strengthen outpatient PC competencies
Ross (2011, USA)	To implement and evaluate a web-based PC training program with competency assessments	Program evaluation with pre/post-tests and faculty assessment metrics. Post-test given after last module before certification	318 residents/fellows (web course) + 18 faculty	Web-based PC course (six modules) + Competency Assessment Tools during clinical rotations	Module pre/post-tests; CAT checklists; faculty assessment tools	Knowledge improved across modules (all post-tests ≥ 80%); CATs feasible for observed skills; faculty assessment scores improved. Demonstrates scalability of web-based PC education combined with structured competency assessment
Gerhardt (2009, USA)	To evaluate a 1-day paediatric PC workshop for oncology fellows with 6- and 12-month follow-up	Pre–post study with follow-ups	32 fellows (26 at 6 month; 22 at 12 month; 20 completed)	1-day workshop covering pain, symptoms, communication, ethics, grief, paediatric-specific issues	Barriers, attitudes, comfort, 32-item knowledge test	Attitudes improved and remained stable; comfort increased by 12 months; overall knowledge improved at post (*p* < 0.001), 6 months (*p* < 0.001), and 12 months (*p* < 0.05). Effective for short-term change but long-term reinforcement needed
Baughcum (2007, USA)	To evaluate a 1-day paediatric PC workshop for oncology fellows	Pre–post study, post study on the same day	32 paediatric oncology fellows	1-day workshop with six modules + parent speakers	Pre/post knowledge + attitudes + beliefs	Knowledge increased significantly (76% → 85%, *p* < 0.001); attitudes improved but discomfort with death persisted. Good immediate gains but indicates need for deeper, longitudinal training

Compared with immersive clinical rotations, non-immersive educational interventions demonstrated a generally smaller magnitude of change across trainee outcomes—a finding consistent with their pedagogical purpose rather than evidence of ineffectiveness. Across this group of studies, four distinct categories emerged: one randomised controlled trial providing the strongest level of evidence, three multi-domain studies assessing improvements across multiple outcomes, two knowledge-only studies demonstrating cognitive gains, and four comfort-only studies focused primarily on perceived preparedness.

The Henselmans randomised controlled trial provides the strongest causal evidence among all non-immersive interventions because it is the only trial using randomisation, the design best suited to determining whether an educational intervention directly produces observable changes in behaviour [25]. Through random allocation, a parallel-group comparison between intervention and control arms, and blinded coding of video-recorded standardised patient encounters using validated observational tools (OPTION12 and 4SDM), the investigators isolated the effect of a 10-h shared decision-making (SDM) training programme from other confounding influences. This rigorous methodology demonstrated large, statistically significant improvements in observed SDM, with scores increasing from 35.6 to 64.0 in the intervention group compared with 34.6 to 44.1 in the control group (*p* value < 0.01). Gains were also seen across all four SDM stages, alongside notable improvements in information sharing and emotion-handling skills during palliative chemotherapy discussions. Overall, the study provides clear evidence that a structured 10-h SDM curriculum can meaningfully enhance observable communication behaviours in palliative chemotherapy encounters.

Within the multi-domain category, studies examined at least two outcomes among knowledge, attitudes, beliefs, or comfort. The study by Gerhardt et al. [26] demonstrated improvements in paediatric fellows’ attitudes and knowledge following a 1-day workshop. Knowledge increased from baseline to post-workshop and remained elevated at both 6 and 12 months, with no significant difference between these follow-up points. Attitudinal improvements emerged between 6 and 12 months; although gains from baseline to 6 months were present, they did not reach statistical significance until the 12-month mark. The longitudinal nature of this study highlights the durability of even a brief educational intervention. Other multi-domain studies showed similar patterns. Chan et al. [27] reported some meaningful gains in comfort and perceived training following a brief multimodality advance care planning curriculum, and Baughcum et al. [28] found that a 1-day workshop improved knowledge, attitudes, and competence across key paediatric palliative care domains, although communication- and ethics-specific gains were more limited. Taken together, these studies indicate that short, structured curricula can address several foundational aspects of palliative care competence.

In contrast, the knowledge-only studies focused exclusively on cognitive outcomes. Nicotra et al. [29] showed that a single 30-min lecture produced substantial knowledge gains (62 to 80%) in differentiating palliative care from hospice care. A notable feature of this study was the associated increase in palliative care consultation activity (from 481 to 778), suggesting that even minimal educational exposure may prompt changes in clinical practice, although durability remains uncertain. Ross et al. [30] evaluated a six-unit web-based palliative care curriculum and demonstrated modest but consistent knowledge improvements following each module, highlighting the scalability of online training formats.

Finally, the comfort-only studies assessed changes in perceived preparedness across different aspects of palliative care following educational interventions. Karlen et al. [31] found that a 90-min resident-led session significantly improved trainees’ comfort across seven domains of palliative care, suggesting feasibility in settings lacking formal palliative care teams. Dewhurst et al. [32] similarly demonstrated increases in comfort across six of seven categories using the CiP-8 tool in a simulation-based package viewed as feasible and useful by trainees. Two smaller studies—Hasan et al. [33] and Salas et al. [34]—reported modest gains in comfort and coping, with Salas et al. also observing an increase in documented Serious Illness Conversations over a 12-month period. However, both interventions were limited by very small sample sizes, restricting the certainty of their conclusions.

Across both immersive and non-immersive interventions, several studies also reported outcomes related to changes in clinical behaviour. These findings are described in detail in the subsequent section on behavioural and practice-related outcomes.

Overall, across these non-immersive interventions, even very brief introductory courses for residents were consistently associated with measurable improvements in knowledge, attitudes, and perceived preparedness, indicating that foundational palliative care competencies can be strengthened through short, structured educational exposure.

### Behavioural and practice-related outcomes

Several studies reported outcomes suggesting potential changes in clinical behaviour following educational interventions, although the strength and nature of this evidence varied. The most robust indications came from studies using direct observational assessments in controlled settings. In particular, a randomised trial evaluating a shared decision-making training programme demonstrated substantial improvements in observed communication behaviours, measured using validated instruments (OPTION12 and 4SDM), indicating behavioural change within simulated palliative chemotherapy consultations [25].

Behavioural change was also reported in immersive educational interventions incorporating objective performance assessments. Studies using observed structured clinical encounters or OSCEs described improvements in communication behaviours, including goals-of-care discussions and end-of-life communication, beyond changes in knowledge or attitudes alone (e.g. Chang et al. [15]; Vergo et al. [24]).

Evidence of translation into routine clinical practice was more limited but present. Two non-immersive studies reported indirect behavioural indicators. Nicotra et al. [29] observed an increase in palliative care consultation activity following a brief educational intervention (from 481 to 778 consultations), and Salas et al. [34] documented an increase in recorded Serious Illness Conversations from 4 to 15.5% over a 12-month period. Several immersive studies also described increased willingness to initiate goals-of-care discussions, greater use of advance care planning tools, and increased confidence in referring patients to specialist palliative care services. However, these outcomes were generally assessed through self-report measures or service-level indicators rather than direct observation of routine clinical practice.

Across studies, behavioural outcomes were typically measured in the short to medium term, ranging from immediate post-intervention assessments to follow-up periods of up to approximately 12 months.

## Discussion

This review identified ten immersive studies and ten non-immersive studies evaluating PC education for oncology residents and fellows, a surprisingly limited evidence base given the importance of palliative competencies in contemporary cancer care. Across immersive interventions, findings generally showed that direct clinical exposure was associated with improvements in trainees’ communication skills, knowledge, attitudes, self-efficacy, and readiness to provide end-of-life care. These findings are encouraging but must be interpreted in the context of predominantly single-group designs, small sample sizes, short-term follow-up, and a heavy reliance on self-reported outcomes. In the absence of control groups in most studies, observed improvements cannot be attributed exclusively to educational intervention. Although most immersive studies originated in North America and varied in methodological rigour, the direction of effect was broadly consistent across settings. This concentration of evidence likely reflects a longer tradition of structured palliative care training and evaluation in North American oncology programmes. For oncology training programmes, this provides reassurance that investing in structured palliative care education—even when delivered in diverse formats and settings—is consistently associated with clinically relevant gains.

Immersive rotations ranged from 2 weeks to 12 months, yet gains did not appear proportional to rotation length. Short rotations of approximately 4 weeks—such as the MD Anderson’s PEACE pathway rotation and Nationwide Hospital’s paediatric programmes—produced consistent improvements in communication, symptom management, and end-of-life preparedness. Intermediate 2-month programmes, such as Chang’s structured curriculum, provided added benefit in objectively assessed humanistic and communication skills. Longer 6- to 12-month rotations also improved knowledge and self-efficacy but did not clearly outperform shorter placements. Together, these findings suggest that a well-designed 4- to 8-week immersive rotation can produce substantial educational benefit and represents a feasible and scalable model for oncology training programmes in Europe. While longer rotations may support deeper professional development and coping skills, current evidence indicates that shorter immersive rotations are sufficient to achieve core palliative care competencies required for oncology practice.

Mandatory rotations, as demonstrated in the studies by Reddy, Vergo, and Chang, showed broader and more consistent improvements than elective placements, likely because they avoid the self-selection bias inherent in elective training. Mandatory programmes also seemed more likely to influence actual clinical behaviour, including referral patterns, indicating greater potential to shape oncology practice.

The strength of evidence varied with evaluation methodology. Studies incorporating objective assessments—such as OSCEs or validated measures of PC knowledge and self-efficacy—generated the most robust findings, whereas those relying solely on self-assessment offered weaker inferential value. Common methodological limitations included small sample sizes, lack of control groups, short-term follow-up, and heavy reliance on subjective outcomes.

Taken as a whole, the strength of evidence underpinning this synthesis is limited. Most included studies used single-group pre–post designs with small samples, typically 12 to 33 residents and relied on self-reported outcomes measured at a single post-intervention time point. Only one randomised controlled trial and two studies with contemporaneous comparison groups were identified. In the absence of control groups, the contribution of general clinical maturation, temporal effects, or Hawthorne effects cannot be excluded. The consistency of direction across studies offers some reassurance; however, the overall certainty of conclusions must be characterised as moderate. Findings should be treated as preliminary and hypothesis-generating, sufficient to inform programme development but not to mandate specific training models.

Non-immersive educational interventions produced smaller and more domain-specific effects than immersive rotations—a finding consistent with their pedagogical purpose rather than evidence of ineffectiveness. Brief courses, workshops, and online modules are well suited to building foundational knowledge, introducing structured communication frameworks, and preparing trainees cognitively for patient-facing clinical contexts. Immersive and non-immersive formats are therefore best understood as complementary strategies targeting different levels of educational outcome, rather than as alternatives in competition. The choice between them—or their deliberate combination—should be guided by the specific competency domain being developed. The strongest evidence came from the randomised controlled trial by Henselmans et al., which demonstrated large, statistically significant improvements in shared decision-making behaviours using validated observational tools, showing that targeted communication-skills training can improve clinical performance independent of immersive experience. Other non-immersive studies showed domain-specific gains: Nicotra et al. demonstrated substantial knowledge improvement and a notable increase in PC consultations in routine practice.

To our knowledge, this is the first review to translate heterogeneous educational interventions into a coherent, tiered model specifically tailored for oncology residency and fellowship programmes.

Importantly, the relevance of these findings must be interpreted considering the competencies already expected of oncologists by international professional standards. As outlined in normative frameworks developed by organisations such as ESMO and articulated in the Lancet Oncology Commission, palliative care competencies—including advanced communication, basic symptom management, timely referral, and interdisciplinary collaboration—are considered core elements of contemporary oncology practice [5, 6]. The present review does not seek to redefine these competencies but rather to examine to what extent existing educational interventions succeed in developing them in practice.

Across the included studies, a core set of competencies was consistently prioritised and assessed. These competencies include advanced communication skills—particularly shared decision-making and goals-of-care discussions—basic symptom management, attitudes towards palliative care, self-efficacy in end-of-life situations, and the appropriate use of palliative care resources, including timely referral. The findings indicate that these competencies can be partially achieved through educational interventions, although evidence of comprehensive and sustained attainment of an integrated palliative care competency profile remains limited, reflecting heterogeneity in study design, outcome measures, and follow-up duration [15, 18, 24, 25, 29]. Importantly, the extent to which these competencies were achieved varied substantially across educational approaches and outcome domains.

Given the heterogeneity of educational interventions, study designs, and outcome measures, formal effect size estimation was not feasible. Nevertheless, when quantitative indicators were reported, immersive clinical rotations were associated with larger absolute changes in outcomes directly linked to clinical practice, such as observed communication behaviours, self-efficacy, and preparedness for end-of-life care (for example, a randomised trial reported an increase in observed shared decision-making scores from 35.6 to 64.0, compared with 34.6 to 44.1 in controls). In contrast, non-immersive interventions tended to produce smaller, more domain-specific effects, most reflected in improvements in knowledge, structured communication frameworks, or self-reported comfort (e.g. short didactic or workshop-based interventions reporting knowledge gains in the range of 15–20 percentage points or modest increases in self-rated comfort). These patterns were consistent across studies despite differences in context, duration, and evaluation methods.

Taken together, the combined evidence indicates that immersive clinical training provides the strongest and most consistent improvements in outcomes directly relevant to oncology practice, while non-immersive approaches remain valuable for foundational knowledge, developing communication frameworks, and preparing trainees for patient-facing experiences. A tiered educational model is therefore supported, in which a mandatory 4- to 8-week immersive PC rotation is complemented by structured non-immersive components that reinforce communication skills, knowledge, and clinical behaviours. Together, this evidence suggests that a tiered model—combining a mandatory immersive rotation with structured non-immersive components—may offer a feasible approach to strengthening PC training within oncology programmes across Europe.

### Limitations

As a rapid review, this synthesis applied methodological adaptations compared with a full systematic review, including restriction of the search to a single database and reliance on narrative synthesis rather than meta-analysis. These adaptations, standard for this review type, may have resulted sometimes in the omission of relevant studies and limit the formal comparability of findings across interventions.

The screening procedure employed a split approach rather than full parallel independent screening of all records by both reviewers. While consistent with accepted rapid review adaptations, this design does not permit the calculation of formal inter-rater agreement statistics such as Cohen’s kappa. This represents a methodological limitation that should be considered when interpreting the reproducibility of the study selection process. In studies without contemporaneous control groups—particularly those with extended follow-up periods—it is not possible to reliably separate the specific effects of the educational intervention from the contribution of general clinical maturation, incidental learning, or concurrent training experiences. This concern is most evident in studies such as Gerhardt et al. [26], where attitudinal improvements reached statistical significance only at 12 months following a 1-day workshop: Alternative explanations, including the accumulating effect of clinical experience, cannot be excluded.

Most studies were also conducted outside Europe, which may reduce generalisability to European oncology training programmes. European systems differ in several relevant respects: the degree to which palliative care is structurally integrated into oncology departments, referral cultures and thresholds for specialist PC involvement, the availability and organisation of specialist PC services, and whether PC training is formally mandated or evaluated within residency curricula. In some European countries, cultural and systemic barriers may result in later and less consistent integration of PC into oncology practice than is observed in North American cancer centres. These differences mean that benefits demonstrated in North American settings cannot be assumed to translate directly to European training environments. Studies conducted within European oncology programmes are urgently needed to provide a context-specific evidence base for the harmonised recommendations that JANE-2 seeks to develop.

Despite these limitations, most studies demonstrated clinically relevant improvements across a range of educational outcomes.

## Conclusions

This rapid review provides the first structured synthesis of educational interventions designed to develop palliative care competencies among oncology residents and fellows. Across the included studies, immersive clinical education—particularly structured rotations of 4 to 8 weeks—was consistently associated with practice-relevant improvements in communication skills, knowledge, attitudes, and self-efficacy. Mandatory formats yielded broader and more consistent benefits than elective placements, reducing self-selection bias and demonstrating greater potential to influence clinical behaviour. Non-immersive interventions contributed meaningful gains in foundational knowledge and structured communication skills and are best understood as complementary to, rather than substitutes for, clinical immersion. Together, these findings suggest that a tiered model—combining a mandatory immersive rotation with structured non-immersive components—may offer a feasible approach to strengthening PC training within oncology programmes.

Important uncertainties remain. The evidence base is predominantly North American, methodologically heterogeneous, and largely reliant on self-reported short-term outcomes. Whether these findings are transferable to European oncology training contexts—where structural, institutional, and cultural conditions differ—cannot be assumed without further research. European studies are a research priority.

Should this model be considered for implementation within oncology training programmes, prospective evaluation would be essential. Key questions yet to be answered include whether short rotations produce sustained competency gains over time; whether observed improvements translate into measurable changes in clinical practice and patient outcomes; and what content, supervision structures, and quality standards are required to ensure effectiveness across diverse training environments.

## Supplementary Information

Below is the link to the electronic supplementary material.

ESM 1 (DOCX 15.0 KB)

## Data Availability

No datasets were generated or analysed during the current study.

## References

[1] Temel JS, Greer JA, Muzikansky A, Gallagher ER, Admane S, Jackson VA, Dahlin CM, Blinderman CD, Jacobsen J, Pirl WF, Billings JA, Lynch TJ (2010) Early palliative care for patients with metastatic non-small-cell lung cancer. N Engl J Med 363(8):733–742. 10.1056/NEJMoa1000678 20818875 10.1056/NEJMoa1000678

[2] Zimmermann C, Swami N, Krzyzanowska M, Hannon B, Leighl N, Oza A, Moore M, Rydall A, Rodin G, Tannock I, Donner A, Lo C (2014) Early palliative care for patients with advanced cancer: A cluster-randomised controlled trial. Lancet Lond Engl 383(9930):1721–1730. 10.1016/S0140-6736(13)62416-2 10.1016/S0140-6736(13)62416-2 24559581

[3] Tripodoro VA, Fidalgo JFL, Pons JJ, Connor SR, Garralda E, Bastos F, Montero Á, Monzón Llamas L, Béjar AC, Suárez D, Centeno C (2025) First-ever global ranking of palliative care: 2025 world map under the new WHO framework. J Pain Symptom Manage 70(5):447–458. 10.1016/j.jpainsymman.2025.07.026 40782892 10.1016/j.jpainsymman.2025.07.026

[4] Schmit JM, Meyer LE, Duff JM, Dai Y, Zou F, Close JL (2016) Perspectives on death and dying: a study of resident comfort with end-of-life care. BMC Med Educ 16(1):297. 10.1186/s12909-016-0819-6 27871287 10.1186/s12909-016-0819-6 PMC5117582

[5] Kaasa S, Loge JH, Aapro M, Albreht T, Anderson R, Bruera E, Brunelli C, Caraceni A, Cervantes A, Currow DC, Deliens L, Fallon M, Gómez-Batiste X, Grotmol KS, Hannon B, Haugen DF, Higginson IJ, Hjermstad MJ, Hui D, Jordan K, Kurita GP, Larkin PJ, Miccinesi G, Nauck F, Pribakovic R, Rodin G, Sjøgren P, Stone P, Zimmermann C, Lundeby T (2018) Integration of oncology and palliative care: A Lancet Oncology Commission. Lancet Oncol 19(11):e588–e653. 10.1016/S1470-2045(18)30415-7 30344075 10.1016/S1470-2045(18)30415-7

[6] Jordan K, Aapro M, Kaasa S, Ripamonti CI, Scotté F, Strasser F, Young A, Bruera E, Herrstedt J, Keefe D, Laird B, Walsh D, Douillard JY, Cervantes A (2018) European Society for Medical Oncology (ESMO) position paper on supportive and palliative care. Ann Oncol Off J Eur Soc Med Oncol 29(1):36–43. 10.1093/annonc/mdx757 10.1093/annonc/mdx757 29253069

[7] Castro JA, Hannon B, Zimmermann C (2023) Integrating palliative care into oncology care worldwide: The right care in the right place at the right time. Curr Treat Options Oncol 24(4):353–372. 10.1007/s11864-023-01060-9 36913164 10.1007/s11864-023-01060-9 PMC10009840

[8] Jordan K, De Azambuja E, Amaral T, Strijbos M, Curigliano G, Lordick F (2023) Medical oncology education in Europe: equipping medical oncologists to provide the best care for patients with cancer. Oncol Res Treat 46(3):72–79. 10.1159/000529128 36642069 10.1159/000529128

[9] Dantigny R, Sanchez S, Tanty A et al (2025) The PALLIA-TRAINING study: mixed-methods study to identify educational needs in palliative care among French oncology fellows. Support Care Cancer 33(8):707. 10.1007/s00520-025-09749-3 40685495 10.1007/s00520-025-09749-3 PMC12277226

[10] Ferrell BR, Temel JS, Temin S, Alesi ER, Balboni TA, Basch EM, Firn JI, Paice JA, Peppercorn JM, Phillips T, Stovall EL, Zimmermann C, Smith TJ (2017) Integration of palliative care into standard oncology care: American Society of Clinical Oncology clinical practice guideline update. J Clin Oncol Off J Am Soc Clin Oncol 35(1):96–112. 10.1200/JCO.2016.70.1474 10.1200/JCO.2016.70.1474 28034065

[11] Tricco AC, Langlois EV, Straus SE (eds) (2017) Rapid reviews to strengthen health policy and systems: a practical guide. World Health Organization, Geneva

[12] Garritty C, Hamel C, Trivella M, Gartlehner G, Nussbaumer-Streit B, Devane D, Kamel C, Griebler U, King VJ, Cochrane Rapid Reviews Methods Group (2024) Updated recommendations for the Cochrane rapid review methods guidance for rapid reviews of effectiveness. BMJ (Clinical research ed.) 384:e076335. 10.1136/bmj-2023-076335 38320771 10.1136/bmj-2023-076335

[13] Page MJ, McKenzie JE, Bossuyt PM et al (2021) The PRISMA 2020 statement: An updated guideline for reporting systematic reviews. BMJ 372:n71. 10.1136/bmj.n71 33782057 10.1136/bmj.n71 PMC8005924

[14] Frank JR, Danoff D (2007) The CanMEDS initiative: implementing an outcomes-based framework of physician competencies. Med Teach 29(7):642–647. 10.1080/01421590701746983 18236250 10.1080/01421590701746983

[15] Chang J, Qi Z, Jiang S, Li L, Sun Q (2021) The impact of palliative care education and training program on the resident physicians. Ann Palliat Med 10(3):2758–2765. 10.21037/apm-20-1625 33549004 10.21037/apm-20-1625

[16] Okon TR, Evans JM, Gomez CF, Blackhall LJ (2004) Palliative educational outcome with implementation of PEACE tool integrated clinical pathway. J Palliat Med 7(2):279–295. 10.1089/109662104773709404 15130206 10.1089/109662104773709404

[17] Olden AM, Quill TE, Bordley D, Ladwig S (2009) Evaluation of a required palliative care rotation for internal medicine residents. J Palliat Med 12(2):150–154. 10.1089/jpm.2008.0246 19207058 10.1089/jpm.2008.0246

[18] Reddy SK, Tanco K, Yennu S et al (2019) Integration of a mandatory palliative care education into hematology-oncology fellowship training in a comprehensive cancer center: a survey of hematology oncology fellows. J Oncol Pract 15(11):e934–e941. 10.1200/JOP.19.00056 31268810 10.1200/JOP.19.00056 PMC7846059

[19] Rossfeld ZM, Tumin D, Humphrey LM (2018) Self-assessment of skills and competencies among residents participating in a pediatric hospice and palliative medicine elective rotation. J Palliat Med 21(2):229–235. 10.1089/jpm.2017.0201 28850307 10.1089/jpm.2017.0201

[20] Biersching T, Schweda A, Oechsle K et al (2023) The OUTREACH study: oncologists of German university hospitals in rotation on a palliative care unit—evaluation of attitude and competence in palliative care and hospice. J Cancer Res Clin Oncol 149(7):2929–2936. 10.1007/s00432-022-04131-w 35831764 10.1007/s00432-022-04131-w PMC10314826

[21] Duong PH, Zulian GB (2006) Impact of a postgraduate six-month rotation in palliative care on knowledge and attitudes of junior residents. Palliat Med 20(5):551–556. 10.1191/0269216306pm1158xx 16903410 10.1191/0269216306pm1158xx

[22] Liao S, Amin A, Rucker L (2004) An innovative, longitudinal program to teach residents about end-of-life care. Acad Med 79(8):752–757. 10.1097/00001888-200408000-00007 15277130 10.1097/00001888-200408000-00007

[23] Centofanti J, Swinton M, Dionne J et al (2016) Resident reflections on end-of-life education: a mixed-methods study of the 3 Wishes Project. BMJ Open 6(3):e010626. 10.1136/bmjopen-2015-010626 27033962 10.1136/bmjopen-2015-010626 PMC4823392

[24] Vergo MT, Sachs S, MacMartin MA, Kirkland KB, Cullinan AM, Stephens LA (2017) Acceptability and impact of a required palliative care rotation with prerotation and postrotation observed simulated clinical experience during internal medicine residency training on primary palliative communication skills. J Palliat Med 20(5):542–547. 10.1089/jpm.2016.0348 27893952 10.1089/jpm.2016.0348

[25] Henselmans I, van Laarhoven HWM, de Haes HCJM et al (2019) Training for medical oncologists on shared decision‐making about palliative chemotherapy: a randomized controlled trial. Oncologist 24(2):259–265. 10.1634/theoncologist.2018-0090 29959285 10.1634/theoncologist.2018-0090 PMC6369949

[26] Gerhardt CA, Grollman JA, Baughcum AE, Young-Saleme T, Stefanik R, Klopfenstein KJ (2009) Longitudinal evaluation of a pediatric palliative care educational workshop for oncology fellows. J Palliat Med 12(4):323–328. 10.1089/jpm.2008.0285 19327067 10.1089/jpm.2008.0285

[27] Chan D, Ward E, Lapin B et al (2016) Outpatient advance care planning internal medicine resident curriculum: valuing our patients’ wishes. J Palliat Med 19(7):734–745. 10.1089/jpm.2015.0313 27244093 10.1089/jpm.2015.0313

[28] Baughcum AE, Gerhardt CA, Young-Saleme T, Stefanik R, Klopfenstein KJ (2007) Evaluation of a pediatric palliative care educational workshop for oncology fellows. Pediatr Blood Cancer 49(2):154–159. 10.1002/pbc.21034 16991132 10.1002/pbc.21034

[29] Nicotra C, Barnes M, Macchio P et al (2021) Educating internal medicine residents on palliative medicine and hospice care at a community teaching hospital. Am J Hosp Palliat Care 38(7):741–744. 10.1177/1049909120979179 33291967 10.1177/1049909120979179

[30] Ross DD, Shpritz DW, Wolfsthal SD et al (2011) Creative solution for implementation of experiential, competency-based palliative care training for internal medicine residents. J Cancer Educ 26(3):436–443. 10.1007/s13187-011-0235-x 21553329 10.1007/s13187-011-0235-x PMC3162123

[31] Karlen N, Cruz B, Leigh AE (2016) Resident-led palliative care education project. J Palliat Med 19(4):428–436. 10.1089/jpm.2015.0246 26859443 10.1089/jpm.2015.0246

[32] Dewhurst F, Howorth K, Billett H et al (2024) Palliative care simulation for internal medicine trainees: development and pilot study. BMJ Support Palliat Care 14(3):358–364. 10.1136/bmjspcare-2021-003272 34531292 10.1136/bmjspcare-2021-003272

[33] Hasan F, Weingarten K, Cada M, Wilejto M (2023) Palliative care training for pediatric hematology/oncology fellows: a Canadian perspective. J Cancer Educ 38(1):167–174. 10.1007/s13187-021-02094-z 34591268 10.1007/s13187-021-02094-z

[34] Salas A, Boanca K, Purdy J et al (2024) Resident-led research: a quality improvement project to improve serious illness conversations. Gerontol Geriatr Educ 45(4):499–504. 10.1080/02701960.2023.2246406 37561638 10.1080/02701960.2023.2246406

