# Supplementary material for: Educational interventions for training oncology residents in palliative medicine: a rapid review of the available evidence to inform training standards in cancer care
Source: Support Care Cancer. 2026 Jun 3;34(6):608. doi: 10.1007/s00520-026-10830-8 (PMC13233664; doi:10.1007/s00520-026-10830-8)
Supplement: Supplementary file 1 — (DOCX 15.0 KB) [file 520_2026_10830_MOESM1_ESM.docx]

**Supplementary Appendix 1. PubMed search strategy**

**Database:** PubMed
**Search date:** 29 September 2025
**Publication date range:** 1 January 2000 to 29 September 2025

("palliative care"[Mesh] OR "palliative medicine"[Mesh] OR "terminal care"[Mesh]

OR "end of life care"[tiab] OR "end-of-life"[tiab] OR "EOL"[tiab]

OR "supportive care"[tiab] OR "serious illness"[tiab] OR palliative[tiab])

AND

("Education, Medical, Graduate"[Mesh] OR "Internship and Residency"[Mesh]

OR "Fellowships and Scholarships"[Mesh] OR education[Mesh] OR teaching[Mesh]

OR curriculum[Mesh] OR "clinical rotation"[tiab] OR "educational intervention"[tiab]

OR simulation[tiab] OR workshop[tiab] OR training[tiab])

AND

("medical oncology"[Mesh] OR "radiotherapy"[Mesh]

OR "radiation oncology"[tiab] OR "clinical oncology"[tiab]

OR "medical oncology"[tiab] OR "hematology oncology"[tiab]

OR "oncology resident"[tiab] OR "oncology residents"[tiab]

OR "oncology fellow"[tiab] OR "oncology fellows"[tiab]

OR "radiation oncology resident"[tiab] OR "radiation oncology residents"[tiab]

OR "specialist registrar"[tiab] OR "registrar"[tiab]

OR ("internal medicine"[Mesh] AND (resident[tiab] OR residents[tiab] OR trainee[tiab]

OR trainees[tiab] OR fellowship[tiab] OR fellow[tiab] OR fellows[tiab])))

AND

(evaluat*[tiab] OR outcome*[tiab] OR effect*[tiab] OR impact[tiab]

OR competence*[tiab] OR skill*[tiab] OR knowledge[tiab]

OR attitude*[tiab] OR "communication skill*"[tiab] OR OSCE[tiab])

NOT

(nurse[tiab] OR nurses[tiab] OR nursing[tiab] OR "family medicine"[tiab]

OR "general practice"[tiab] OR "primary care"[tiab] OR pharmacy[tiab]

OR pharmacist[tiab] OR "physician assistant"[tiab] OR "medical student"[tiab]

OR undergraduate[tiab] OR dental[tiab] OR midwife[tiab] OR midwives[tiab]

OR paramedic[tiab] OR "social work"[tiab] OR psychology[tiab])

AND

("2000/01/01"[Date - Publication]: "2025/09/29"[Date - Publication])
